# Carbon Ion Radiation Therapy for Nonmetastatic Castration-Resistant Prostate Cancer: A Retrospective Analysis

**DOI:** 10.1016/j.adro.2023.101432

**Published:** 2023-12-30

**Authors:** Yuhei Miyasaka, Hidemasa Kawamura, Hiro Sato, Nobuteru Kubo, Hiroyuki Katoh, Hitoshi Ishikawa, Hiroshi Matsui, Yoshiyuki Miyazawa, Kazuto Ito, Kazuhiro Suzuki, Tatsuya Ohno

**Affiliations:** a Department of Radiation Oncology, Gunma University Graduate School of Medicine, Showa-machi, Maebashi, Gunma, Japan; b Gunma University Heavy Ion Medical Center, Showa-machi, Maebashi, Gunma, Japan; c Department of Radiation Oncology, Kanagawa Cancer Center, Nakao, Asahi-ku, Yokohama, Kanagawa, Japan; d QST Hospital, National Institutes for Quantum Science and Technology, Anagawa, Inage-ku, Chiba, Chiba, Japan; e Department of Urology, Gunma University Graduate School of Medicine, Showa-machi, Maebashi, Gunma, Japan

## Abstract

**Purpose:**

Treatment outcomes of definitive photon radiation therapy for nonmetastatic castration-resistant prostate cancer (nmCRPC) are reportedly unsatisfactory. Carbon ion radiation therapy (CIRT) has shown favorable tumor control in various malignancies, including radioresistant tumors. Therefore, we retrospectively evaluated the clinical outcomes of CIRT for nmCRPC.

**Methods and Materials:**

Patients with nmCRPC (N0M0) treated with CIRT at a total dose of 57.6 Gy (relative biologic effectiveness) in 16 fractions or 51.6 Gy (relative biologic effectiveness) in 12 fractions were included. The castration-resistant status received a diagnosis based on prostate-specific antigen kinetics showing a monotonic increase during primary androgen deprivation therapy or the need to change androgen deprivation therapy. Clinical factors associated with patient prognosis were explored. Twenty-three consecutive patients were identified from our database. The median follow-up period was 63.6 months (range, 14.1-120).

**Results:**

Seven patients developed biochemical relapse, 6 had clinical relapse, and 4 died of the disease. The 5-year overall survival, local control rate, biochemical relapse-free survival, and clinical relapse-free survival were 87.5%, 95.7%, 70.3%, and 75.7%, respectively. One patient with diabetes mellitus requiring insulin injections and taking antiplatelet and anticoagulant drugs developed grade 3 hematuria and bladder tamponade after CIRT. None of the patients developed grade 4 or worse toxicity.

**Conclusions:**

The present findings indicate the acceptable safety and favorable efficacy of CIRT, encouraging further research on CIRT for nmCRPC.

## Introduction

Radiation therapy is a standard treatment for nonmetastatic prostate cancer.^1^ Androgen deprivation therapy (ADT) is also a common treatment for prostate cancer; however, it may cause castration-resistant prostate cancer (CRPC), which is life-threatening. Previous studies have evaluated the effectiveness of photon radiation therapy for nonmetastatic CRPC (nmCRPC), showing 5-year biochemical relapse-free survival (BRFS), clinical relapse-free survival (CRFS), and overall survival (OS) rates of less than 40%, 60%, and 75%, respectively.^2^, ^3^, ^4^, ^5^, ^6^, ^7^ These outcomes are unsatisfactory compared with those of definitive radiation therapy for nonmetastatic hormone-sensitive prostate cancer, which has shown a 5-year BRFS, CFRS, and OS of approximately 80 to 90%, 90%, and 100%, respectively.^8^, ^9^, ^10^ Therefore, improvements in radiation therapy for nmCRPC are necessary.

Carbon ion radiation therapy (CIRT) refers to external beam radiation therapy using carbon ions. CIRT has biophysical advantages over photon radiation therapy, such as a stronger cell-killing ability per physical dose and better dose distribution owing to the Bragg peak.^11^ Several prospective studies have reported on the safety and effectiveness of CIRT for nonmetastatic prostate cancer. Moreover, CIRT appears to be as effective in high-risk prostate cancer as it is in low- and intermediate-risk, with a 5-year BRFS of approximately 90%.^12^, ^13^, ^14^, ^15^, ^16^, ^17^ Therefore, CIRT may also be effective for nmCRPC. Thus, we retrospectively analyzed patients with nmCRPC (N0M0) treated with CIRT to evaluate the effectiveness of CIRT for nmCRPC.

## Methods and Materials

### Study design

This study was conducted according to the principles outlined in the Declaration of Helsinki after receiving approval from the ethics review board of Gunma University (HS2020-021). The requirement for written informed consent was waived because of the retrospective observational nature of this study, but all participants or their relatives were given the opportunity to opt-out.

The inclusion criteria were mainly based on those used in a previous study on photon radiation therapy for CRPC by Aizawa et al.^7^ The inclusion criteria were as follows: (1) pathologic diagnosis of prostate cancer by a central pathologist; (2) clinical diagnosis of N0M0 according to the International Union Against Cancer TNM classification (2002); (3) serum prostate-specific antigen (PSA) kinetics showing a monotonic increase during primary ADT or need for ADT modifications; and (4) treatment with CIRT at Gunma University Heavy Ion Medical Center between March 2010 and December 2019. Since CIRT was administered for localized untreated disease in the clinical practice, patients who underwent radical prostatectomy or radiation therapy to the prostate in the hormone-sensitive stage were not included in the present study. Patients who refused regular follow-up before the CIRT were excluded.

Clinical staging was based on computed tomography (CT), magnetic resonance imaging (MRI), and bone scintigraphy findings at the initiation of the primary ADT. Pathologic grades were assigned according to the modified Gleason grading system proposed by the International Society of Urological Pathology.^18^ Before CIRT, CT, MRI, and bone scintigraphy were performed for screening of metastasis.

### CIRT

CIRT was administered once a day, with 4 sessions a week. The patients were positioned in a customized cradle with a low-temperature thermoplastic sheet. Treatment planning was performed using XiO-N (Elekta and Mitsubishi Electric) with a set of scans with 2-mm-thick CT slices fused with MRI. For irradiation, a spread-out Bragg peak was used with multileaf collimators and compensation bolus for each patient. All treatment plans were approved by the institutional conference, and written informed consent was obtained from each patient before treatment.

Dose fractionations and planning were different between March 2010 to November 2016 and those thereafter.

Between March 2010 and November 2016, a total dose of 57.6 Gy (relative biologic effectiveness [RBE]) was administered in 16 fractions over 4 weeks, with a fractional dose of 3.6 Gy (RBE). The details have been reported previously.^16^ The clinical target volume (CTV) included gross tumor volume, whole prostate, and proximal seminal vesicles. The primary planning target volume (PTV1) for the initial 9 fractions was generated by adding 10 mm anterior and lateral margins, 6 mm cranial and caudal margins, and a 5 mm posterior margin to the CTV, and 3 mm lateral margins to seminal vesicle. The second PTV (PTV2) for the latter 7 fractions was generated by excluding the posterior, cranial, and caudal PTV margins from the PTV1. Three radiation ports were used in the bilateral and anterior directions.

After November 2016, the prescribed dose was set to 51.6 Gy (RBE) in 12 fractions over 3 weeks, with a fractional dose of 4.3 Gy (RBE). The CTV was the same as that before November 2016. The PTV1 for the initial 8 fractions included the CTV, anterior and lateral margins of 8 mm, cranial and caudal margins of 6 mm, posterior margin of 5 mm, and lateral margins to the seminal vesicle of 3 mm. The PTV2 for the latter 4 fractions was generated by the same method as those before. The radiation ports were used in the bilateral direction.

### ADT after CIRT

All patients received ADT. Since an optimal duration of ADT for nmCRPC has not been established, it was based on the discretion of the urologic physicians (Table E1). Some patients also received second-generation antiandrogens, such as enzalutamide and abiraterone.

### Follow-up evaluations

The follow-up period was calculated from the first date of irradiation. All included patients were followed up at 3- to 6-month-intervals for 5 years and at 6- to 12-month-intervals thereafter. Physical, blood, including serum PSA, and urine examinations were performed at each follow-up. CT, MRI, bone scintigraphy, and transrectal ultrasonography were performed at least once a year for 5 years in principle.

The efficacy endpoints were OS, prostate cancer-specific survival (PCSS), LC, BRFS, and CRFS. Biochemical relapse was defined according to the Radiation Therapy Oncology Group-Association of Therapeutic Radiation Oncology Phoenix Consensus Conference definition (nadir + 2.0 ng/mL).^19^ Clinical relapse was defined as relapse diagnosed by imaging. Local relapse was defined as clinical relapse in the prostate or irradiated seminal vesicles. Adverse events were evaluated according to the National Cancer Institute Common Terminology Criteria for Adverse Events (version 5.0).

### Statistical analyses

OS, PCSS, LC, BRFS, and CRFS were estimated using Kaplan-Meier methods. Differences in survivals between subgroups stratified by clinical factors were evaluated using log-rank tests. A *P* value less than .05 was considered statistically significant. All statistical analyzes were performed using R version 3.6.2.^20^

## Results

### Patient characteristics

Twenty-three consecutive patients meeting the inclusion criteria were identified in our database. The patients’ characteristics are summarized in Table 1. The median follow-up period was 63.6 months (range, 14.5-120). Although administered ADT was not uniform, all patients were treated with luteinizing hormone-releasing hormone agonists or antagonists at least for 6 months before CIRT. Seven patients received lifelong ADT, while the remaining 16 patients received ADT for a median duration of 41 months (range, 8-102). Details of the ADT are summarized in Table E1.

**Table 1:** Summary of characteristics of the included patients (n = 23)

Characteristic	Median (range); n (%)
Age, y	72 (52-88)
Initial PSA, ng/mL	13.8 (4.91-96.2)
Nadir* PSA, ng/mL	0.30 (<0.01-14.6)
PSA before CIRT, ng/mL	1.32 (<0.01-25.8)
Age, y	72 (52-88)
T stage at diagnosis	
1c	4 (17%)
2a	2 (8.7%)
2b	1 (4.3%)
2c	1 (4.3%)
3a	11 (48%)
3b	3 (13%)
4	1 (4.3%)
Gleason grade	
2	7 (30%)
3	4 (17%)
4	5 (22%)
5	7 (30%)
NCCN risk	
Favorable intermediate	1 (4.3%)
Unfavorable intermediate	2 (8.7%)
High	10 (43%)
Very high	9 (39%)
Dose, Gy (RBE)	
51.6	9 (39%)
57.6	14 (61%)
ADT duration before CIRT, mo	37.0 (6.00-189)

*Abbreviations*: ADT = androgen deprivation therapy; CIRT = carbon ion radiation therapy; NCCN = National Cancer Comprehensive Network; PSA = prostate-specific antigen; RBE = relative biologic effectiveness.

⁎ Nadir is defined as the lowest value of PSA during the primary ADT.

### Clinical outcomes

**Figure 1:**
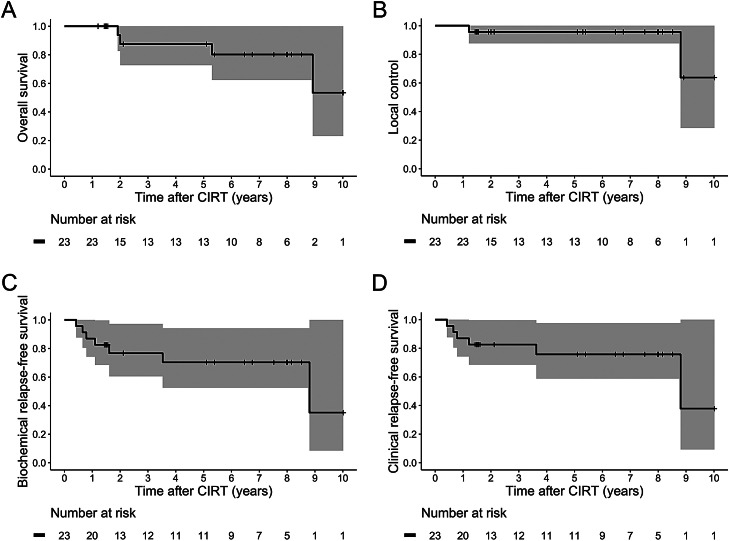
Kaplan-Meir curves in 23 patients. (A) Overall survival (equal to prostate cancer-specific survival), (B) local control, (C) biochemical relapse-free survival, and (D) clinical relapse-free survival. The gray regions show 95% confidence intervals. *Abbreviation*: CIRT = carbon ion radiation therapy.

Of the 23 patients, 4 died of CRPC. No patient died of other causes. Biochemical relapse was observed in 7 patients. Clinical relapse was observed in 6 patients, including 3 patients with multiple sites. The site of relapse was as follows: prostate in 2 patients; bone in 4; lymph node in 3; lungs in one; and meninges in one (Table E1). Kaplan-Meir curves of OS (equal to PCSS), LC, BRFS, and CRFS are shown in Fig. 1. The OS, LC, BRFS, and CRFS were as follows: 3-year, 87.5% [95% CI, 72.7%-100%], 95.7% (95% CI, 87.7%-100%), 76.7% (95% CI, 60.5%-97.2%), and 82.6% (95% CI, 68.5%-99.6%), respectively; 5-year, 87.5% (95% CI, 72.7%-100%), 95.7% (95% CI, 87.7%-100%), 70.3% (95% CI, 52.5%-94.2%), and 75.7% (95% CI, 58.8%-97.6%), respectively; 8-year, 80.2% (95% CI, 64.2%-100%), 95.7% (95% CI, 87.7%-100%), 70.3% (95% CI, 52.5%-94.2%), and 75.7% (95% CI, 58.8%-97.6%), respectively. The toxicities developing after CIRT are summarized in Table 2. There was no grade 3 or worse acute toxicity. As for late toxicities, one patient with diabetes mellitus needing insulin injections and taking antiplatelet and anticoagulant drugs developed grade 3 hematuria and bladder tamponade. There was no grade 4 or worse late toxicity. Grade 2 late urinary disorders were observed in 17% (4/23) of the patients, and grade 2 late rectal bleeding was observed in 4.3% (1/23).

**Table 2:** Summary of toxicities (n = 23)

Toxicities		Genitourinary n (%)	Gastrointestinal n (%)
Acute	Grade 0	6 (26)	23 (100)
	Grade 1	13 (57)	0 (0)
	Grade 2	4 (17)	0 (0)
	Grade 3≤	0 (0)	0 (0)
Late (maximum)	Grade 0	12 (52)	21 (91)
	Grade 1	8 (35)	1 (4.3)
	Grade 2	4 (17)	1 (4.3)
	Grade 3	1 (4.3)	0 (0)
	Grade 4≤	0 (0)	0 (0)

### Associations of clinical parameters with outcomes

The associations of age, T stage at diagnosis, initial PSA, PSA at nadir during primary ADT, PSA at CIRT, Gleason grade group, and ADT period before CIRT with OS and BRFS were evaluated using a log-rank test (Table 3). No significant difference was observed among these factors, but there were some trends indicating favorable OS, BRFS, and CRFS in patients with a lower PSA value at CIRT (*P* = .23, .14, and .26, respectively); favorable BRFS and CRFS in patients with T1c-2c disease (*P* = .16 and .25, respectively); and favorable CRFS in patients with Gleason grade group 2 to 4 (*P* = .16).

**Table 3:** Summary of log-rank tests for overall survival and biochemical relapse-free survival (n = 23)

		OS		BRFS		CRFS	
Parameters	n	5-y (%)	*P* value	5-y (%)	*P* value	5-y (%)	*P* value
Age, y							
≤72	12	90.0	.74	75.0	0.87	75.0	.52
>72	11	83.3		56.8		68.2	
T stage, ng/mL							
1c-2c	8	85.7	.55	100	0.16	87.5	.25
3a-4	15	88.9		58.3		66.7	
Initial PSA, ng/mL							
≤13.8	12	87.5	.47	71.4	0.98	83.3	.68
>13.8	11	87.5		70.1		70.1	
PSA at nadir, ng/mL*							
≤0.30	12	88.9	.99	65.6	0.82	75.0	.92
>0.30	11	85.7		72.7		72.7	
PSA at CIRT, ng/mL							
≤1.3	12	100	.23	83.3	0.14	83.3	.26
>1.3	11	71.4		51.1		61.4	
Gleason grade group							
2-4	11	100	.50	72.9	0.36	83.3	.16
5	12	77.8		66.7		66.7	
ADT period before CIRT, mo							
≤37	12	80.0	.56	75.0	0.85	75.0	.76
>37	11	100		56.8		68.2	

*Abbreviations*: ADT = androgen deprivation therapy; BRFS = biochemical relapse-free survival; CIRT = carbon ion radiation therapy; CRFS = clinical relapse-free survival; OS = overall survival; PSA = prostate-specific antigen.

⁎ Nadir is defined as the lowest value of PSA during the primary ADT.

## Discussion

To the best of our knowledge, this is the first report on the clinical outcomes of CIRT for nmCRPC. The 5-year OS, LC, BRFS, and CRFS observed in this study were 87.5%, 95.7%, 70.3%, and 75.7%, respectively. Our findings demonstrate favorable outcomes with tolerable toxicities compared with those in previous studies on photon radiation therapy (Table 4). Thus, CIRT seems to be a promising local treatment option for nmCRPC.

**Table 4:** Comparison of clinical outcomes after radiation therapy for nmCRPC

										Probability (5-y)				
Author, year		N	Modality	Gleason score >7 (%)	N1/ NX (%)	iPSA, (ng/mL, median)	Pre-RT PSA (ng/mL, median)	Total dose	Follow-up (mo, median)	OS	PCCS	BRFS	CRFS	LC
Akimoto et al,^2^ 2004		53	Photon	47	0/ 0	NA	9.7*^,^	69 Gy	35	-	87	-	56	-
Sanguineti et al,^3^ 2004		29	Photon	38	15/ 0	15	7.4	66-76 Gy	33	28	-	-	-	89
Ogawa et al,^4^ 2009		84	Photon	NA	12/ 19	9.7	NA	30-76 Gy†	27	67 (3y)	-	61 (3y)	-	93 (3y)
Sasaki et al,^5^ 2009		140	Photon	NA	22/ 5	35	10	66 Gy‡	21	48	-	-	37	-
Botticella et al,^6^ 2013		42	Photon	29	0/ NA	16	3.7	78 Gy	53	65	65	39.4	60	100
Aizawa, et al,^7^ 2018		31	Photon	32	0/ 0	34	2.0	66-78 Gy§	67	74.6	77.4	32.3	56.0	91.0
Present 2020		23	Carbon	52	0/ 0	14	1.3	57.6 Gy (RBE)	64	87.5	87.5	70.3	75.7	95.7

*Abbreviations*: BRFS = biochemical relapse-free survival; CRFS = clinical relapse-free survival; iPSA = initial prostate-specific antigen; LC = local control rate; NA = not available; nmCRPC = nonmetastatic castration-resistant prostate cancer; OS = overall survival; PCCS = prostate cancer-specific survival; pre-RT-PSA = pre radiation therapy prostate-specific antigen; RBE = relative biologic effectiveness.

⁎ Average

† Median 66 Gy.

‡ 50% received pelvic irradiation.

§ 22.6% received pelvic irradiation plus prostate boost.

There are few studies on definitive radiation therapy for nmCRPC. Aizawa et al observed 5- and 8-year BRFS of 32.3% (95% CI, 16.9%-48.6%) and 25.8% (95% CI, 12.2%-41.8%), respectively, after photon radiation therapy.^7^ Botticella et al reported 3- and 5-year BRFS of 60.2% (95% CI, 42.6%-73.9%) and 39.4% (95% CI, 22.7%-55.7%), respectively. In comparison, our findings of 3-, 5-, and 8-year BRFS of 76.7% (95% CI, 60.5%-97.2%), 70.3% (95% CI, 52.5%-94.2%), and 70.3% (95% CI, 52.5%-94.2%), respectively, seem favorable. The differences in these findings could be explained by the different antitumor effects of CIRT and x-ray radiation therapy. An in vitro study has suggested that irradiation doses required to kill 50% of the tumor cell population were significantly higher for androgen-resistant prostate cancer cells than for androgen-sensitive prostate cancer cells on using x-rays, with no such difference observed on using carbon ion beams.^21^

The favorable CRFS observed in the present study is also noteworthy. Recent phase 3 clinical trials have demonstrated that second-generation antiandrogens, such as enzalutamide, apalutamide, and darolutamide, have significantly improved metastasis-free survival in N0M0 CRPC, with a median metastasis-free survival of 40.5 months observed in the SPARTAN trial^22^ and 36.3 months in the PROSPER trial.^23^ In our study, 5-year CRFS of 75.7% was found after CIRT. Furthermore, only 6 (26%) patients developed clinical relapse despite administering CIRT only to the prostate and seminal vesicle, whereas in another study, metastasis was detected by prostate-specific membrane antigen-positron emission tomography/CT in 55% of patients with nmCRPC diagnosed by CT/MRI/bone scintigraphy.^24^ Although the reason for this outcome was not determined in this study, peripheral immune activation after CIRT may be responsible.^25^ Despite differences in background and evaluation methods between the previous studies and our study, our findings indicate that CIRT could be a promising treatment option for nmCRPC. Additional studies validating our results with a longer follow-up are necessary. In addition, the combination of CIRT with second-generation antiandrogens seems to be worth exploring.

We also evaluated associations between clinical factors and oncological outcomes. Lower PSA levels at CIRT, T1c-2c disease, and Gleason grade group 2 to 4 showed a trend toward favorable prognoses; however, there was no significant difference in the prognoses. Although no such tendency was observed here, several studies on photon radiation therapy have shown that lower PSA levels at nadir indicate favorable prognoses.^4^^,^^6^^,^^7^ The present study could not identify potential prognostic factors that may be the subject of further research.

No severe toxicity was observed in the present study. One patient developed grade 3 late hematuria and bladder tamponade; however, this complication may have partially been affected by anticoagulant and antiplatelet use accompanied by diabetes mellitus requiring insulin injection. Grade 2 late genitourinary and gastrointestinal toxicities were observed in 17% (4/23) and 4.3% (1/23) of the patients. The incidence of late toxicities in this study was slightly higher than previously reported,^12^, ^13^, ^14^, ^15^, ^16^, ^17^ which may be partially attributed to our small sample. However, the observed toxicities were manageable and thus considered acceptable.

Although discussing CRPC, its definition should also be considered. The Prostate Cancer Clinical Trials Working Group defined CRPC as prostate cancer that progresses clinically, radiographically, or biochemically despite castrate levels of serum testosterone (<50 ng/dL). This definition was advocated for clinical trials evaluating systemic treatments for prostate cancer.^5^ In contrast, previous observational studies on photon radiation therapy for nmCRPC have used different definitions of CRPC that were either not based on serum testosterone levels or were less strict. In the present study, because data on serum testosterone levels were insufficient, we defined CRPC as prostate cancer with serum PSA kinetics showing a monotonic increase during primary ADT or the need for modification of ADT. This definition was like that used in a previous study on photon radiation therapy.^7^

This study had some limitations. Potential sources of bias exist because of the retrospective design and small sample. Besides, no control cohort was included. Therefore, future comparative studies are needed to confirm the clinical superiority of CIRT. The ADT regimens may have also affected patient prognoses. Future studies using a protocol-based ADT are warranted.

## Conclusion

In summary, our findings indicate that CIRT is a promising modality for the management of nmCRPC. Although the present study may not provide robust evidence, our findings encourage further research on the use of CIRT for nmCRPC.

## Disclosures

Kazuhiro Suzuki has potential financial conflicts of interest due to consultancies in Takeda Pharmaceutical, Astellas Pharma, Daiichi-Sankyo, AstraZeneca, Sanofi, Janssen, and Bayer and grants received from Takeda Pharmaceutical, Astellas Pharma, and Daiichi-Sankyo. Tatsuya Ohno has potential financial conflicts of interest due to research funding from Hitachi Ltd Hiroyuki Katoh has potential financial conflicts of interest due to research funding from Toshiba Energy Systems & Solutions Corporation.

## Appendix Supplementary materials

Supplementary Table Table A1. Summary for ADT of the included patients and the outcomes Image, application 1

## Footnotes

Supplementary material associated with this article can be found in the online version at doi:10.1016/j.adro.2023.101432.
