# Supplementary material for: Carbon Ion Radiation Therapy for Nonmetastatic Castration-Resistant Prostate Cancer: A Retrospective Analysis
Source: Adv Radiat Oncol. 2023 Dec 30;9(4):101432. doi: 10.1016/j.adro.2023.101432 (PMC11110035; doi:10.1016/j.adro.2023.101432)
Supplement: Supplementary Table — Table A1. Summary for ADT of the included patients and the outcomes [file mmc1.docx]

**Table A1. Summary for ADT of the included patients and the outcomes**

| No. | Total ADT (months) | ADT before CIRT (months) | Primary ADT | 2^nd^ generation antiandrogen^1^ before CIRT | Disease Progression after CIRT | Relapse Regions | Salvage 2^nd^ generation^1^ antiandrogen |
| --- | --- | --- | --- | --- | --- | --- | --- |
| 1 | 50 | 27 | MAB^2^ | No | No | - | - |
| 2 | 28 | 7 | LH-RH | No | Yes | Prostate | No |
| 3 | 78 | 60 | LH-RH | No | No | - | - |
| 4 | 165 | 165 | MAB | No | Yes | Bone | No |
| 5 | Lifelong^3^ | 36 | MAB | No | Yes | Lymph node | No |
| 6 | Lifelong^4^ | 138 | LH-RH | No | No | - | - |
| 7 | 8 | 7 | LH-RH | No | No | - | - |
| 8 | 56 | 36 | MAB | No | No | - | - |
| 9 | 30 | 10 | MAB | No | No | - | - |
| 10 | 32 | 16 | LH-RH | No | No | - | - |
| 11 | 47 | 47 | MAB | No | No | - | - |
| 12 | 73 | 69 | MAB | No | Yes | Bone, Lung, Meninges | Yes |
| 13 | 25 | 7 | LH-RH | No | No | - | - |
| 14 | 85 | 26 | MAB | No | No | - | - |
| 15 | Lifelong^3^ | 12 | LH-RH | No | Yes | Bone, Lung, Lymph node | Yes |
| 16 | Lifelong^4^ | 83 | LH-RH | No | No | - | - |
| 17 | 102 | 87 | LH-RH | No | No | - | - |
| 18 | 41 | 37 | LH-RH | No | No | - | - |
| 19 | Lifelong^4^ | 73 | LH-RH | No | No | - | - |
| 20 | Lifelong^3^ | 114 | MAB | Yes^5^ | Yes | Not detected | Yes |
| 21 | Lifelong^4^ | 78 | LH-RH | No | No | - | - |
| 22 | 41 | 41 | MAB | No | No | - | - |
| 23 | 23 | 11 | MAB | No | Yes | Prostate, Bone | No |

^1^ 2^nd^ generation antiandrogen: enzalutamide or abiraterone

^2^ LH-RH plus antiandrogen

^3^ Lifelong ADT due to the disease progression during adjuvant ADT after CIRT

^4^ Planned lifelong ADT

^5^ Enzalutamide was administered but terminated before CIRT because of fatigue.

Abbreviations: ADT, androgen deprivation therapy; CIRT, carbon ion radiotherapy; LH-RH, luteinizing hormone-releasing hormone agonist/ antagonist monotherapy; MAB, maximum androgen blockade
